# Distinct profile and prognostic impact of body composition changes in idiopathic pulmonary fibrosis and idiopathic pleuroparenchymal fibroelastosis

**DOI:** 10.1038/s41598-018-32478-z

**Published:** 2018-09-19

**Authors:** Yuzo Suzuki, Katsuhiro Yoshimura, Yasunori Enomoto, Hideki Yasui, Hironao Hozumi, Masato Karayama, Kazuki Furuhashi, Noriyuki Enomoto, Tomoyuki Fujisawa, Yutaro Nakamura, Naoki Inui, Takafumi Suda

**Affiliations:** Second Division, Department of Internal Medicine, Hamamatsu University School of Medicine, Hamamatsu, Japan

## Abstract

Change in body composition with skeletal muscle wasting, a major component of pulmonary cachexia, is associated with mortality in chronic obstructive pulmonary disease and cancer. However, its relevance in interstitial lung diseases (ILDs) remains unclear. We hypothesized changes in body composition would be associated with mortality in ILDs. We measured the cross-sectional-area (ESM_CSA_) and muscle attenuation (ESM_MA_) of erector-spinae muscles, as determined by CT-imaging, in patients with idiopathic pulmonary fibrosis (IPF; n = 131) and idiopathic pleuroparenchymal fibroelastosis (iPPFE; n = 43) and controls. Subsequently, implications with prognosis were evaluated. The ESM_CSA_ of ILD patients, but not ESM_MA_, was significantly smaller than that in controls. Lower ESM_CSA_ with decreased BMI were recorded in iPPFE patients versus IPF patients, whilst IPF patients had decreased ESM_CSA_ without BMI decline. Lower ESM_CSA_ in IPF patients were associated with poorer prognoses. Conversely, decreased ESM_MA_ were associated with worse survival in iPPFE patients. Multivariate analyses showed that ESM_CSA_ in IPF and ESM_MA_ in iPPFE were independent risk factors for mortality. Distinct changes in body composition had prognostic significance among patients with IPF and iPPFE. Lower ESM_CSA_ and ESM_MA_ were independently associated with poor prognosis in IPF and iPPFE, respectively. These results suggest values to measure body composition changes in managing patients with IPF and iPPFE.

## Introduction

Cachexia is a complex metabolic syndrome and occurs in association with an underlying illness. It is characterized by loss of muscle, with or without loss of fat mass^1,2^. Cachexia and muscle wasting cause changes of body composition and are recognized as common features of advanced respiratory disease. To date, cachexia has been most extensively studied in patients with chronic obstructive pulmonary disease (COPD) and cancer. Cachexia in cancer is associated with reduced physical function^3^, poor tolerance to anti-cancer therapies^4^, decreased quality of life^5^, and poorer prognoses^6^. Similarly, muscle wasting in COPD, in which both cachexia and sarcopenia are also partly involved, contributes to diminished skeletal muscle function, reduced exercise capacity, and decreased health status^7,8^. It is also a determinant of mortality in COPD, independent of airflow obstruction^9,10^.

Dual-energy X-ray absorption (D-XA) has been traditionally employed to assess changes in body composition^11^. However, recently, advances in imaging techniques have enabled the quantitative assessment of skeletal muscle loss on computed tomography (CT). CT-derived cross-sectional skeletal muscle area analyses are powerful predictors of survival, superior to body mass index (BMI), in patients with both cancer and COPD^12–14^.

Pulmonary fibrosis is a chronic, frequently progressive, fibrosing interstitial lung disease (ILD) with few therapeutic options. Among the various ILDs, idiopathic pulmonary fibrosis (IPF) is the most common type of idiopathic interstitial pneumonia. It is defined as a specific form of chronic, progressive fibrosis, with a basal predominance^15–17^. Conversely, idiopathic pleuroparenchymal fibroelastosis (iPPFE) is a rare ILD that was newly defined with characteristic histological features in the European Respiratory Society (ERS)/American Thoracic Society (ATS) guideline^15^. Compared to the lower-lobe dominant IPF, iPPFE is characterized by upper lobe predominant fibrosis involving the pleura and subpleural lung parenchyma^15^. These distinct types of ILDs cause progressive dyspnea and lung function decline, resulting in pulmonary dysfunction and cachexia syndrome. Body composition changes are typically found in iPPFE; patients often complain of weight loss associated with a slender stature, with a “flattened chest” being a unique peculiar physical finding^18,19^. However, despite their occurrence, the clinical implications of body composition changes in ILDs are currently unclear.

Thus, we hypothesized that changes in body composition would be associated with mortality in ILDs. In this study, we aimed to compare the cross-sectional area (ESM_CSA_) and muscle attenuation (ESM_MA_) of erector spine muscle, as determined by CT-imaging, in patients with IPF and iPPFE. We also aimed to explore the impact of body composition changes on prognoses in patients with IPF and iPPFE.

## Results

### Clinical characteristics

The clinical characteristics of 131 patients with IPF and 43 patients with iPPFE are summarized in Table 1 and Supplement Table 1. Among the 131 patients with IPF, 81 patients (61.8%) were diagnosed with IPF by the presence of usual interstitial pneumonia pattern on high-resolution CT images, and 50 patients (38.2%) were histologically diagnosed with IPF via surgical lung biopsies. As expected, there were several differences between the patients with IPF and iPPFE; there were more male smokers and levels of serum Krebs von den Lungen-6 (KL-6) were increased in the IPF group, whilst patients with iPPFE had lower BMIs (median 17.2 [14.7–18.5] kg/m^2^), occasional “flattened chest” as previously described^18,20,21^, lower % forced vital capacity (FVC)-predicted, and higher partial pressures of carbon dioxide (PaCO_2_). The levels of serum KL-6 were increased in patients with IPF compared to those in iPPFE patients. Patients with IPF and iPPFE did not differ in terms of predicted values of diffusing capacity of the lung for carbon monoxide (DLCO; n = 52 and n = 24, respectively) or serum levels of albumin and surfactant protein-D (SP-D).

**Table 1:** Clinical characteristics of 131 IPF patients and 43 iPPFE patients.

	IPF (n = 131) UIP/IPF 50 (38.2%), cIPF 81(61.8%)	iPPFE (n = 43)
Age, yr	69.0 [64.0–75.0]	69.0 [64.0–74.0]
Sex, male/female	117 (89.3%)/14 (10.7%)	27 (62.8%)/16 (37.2%)
Observation period, mo	53.3 [31.6–86.1]	31.3 [18.2–47.2]
Never or former/current smoker	20 (15.3%), 111 (84.7%)	29 (67.4%), 14 (32.6%)
Smoking pack-year	35.0 [20.0–60.0]	0 [0–12.5]
acute exacerbation, yes	39 (29.8%)	8 (18.6%)
Height, cm	162.1 [157.0–166.0]	159.0 [152.0–165.0]
Weight, kg	60.0 [52.1–67.0]	42.3 [35.8–48.0]
BMI, kg/m^2^	23.1 [21.3–24.7]	17.2 [14.7–18.5]
ESM_CSA,_ cm^2^	32.8 [27.1–37.7]	23.4 [17.8–30.6]
ESM_MA_, HU	42.0 [35.7–45.7]	43.5 [38.2–48.7]
**Pulmonary Function Test**		
FVC, %-pred	80.5 [66.4–92.9] (n = 120)	54.4 [45.8–67.5] (n = 37)
FEV_1_/FVC, %	83.5 [79.4–88.0] (n = 120)	96.3 [91.4–100] (n = 37)
DLCO, %	68.6 [55.4–97.1] (n = 52)	68.7 [47.9–91.9] (n = 24)
**Laboratory**		
PaO_2_, Torr	80.0 [73.0–89.1] (n = 111)	79.0 [71.5–84.9] (n = 38)
PaCO_2_, Torr	41.9 [39.0–44.0] (n = 111)	46.7 [41.3–49.0] (n = 38)
Alb, g/dl	4.0 [3.8–4.3] (n = 124)	4.0 [3.5–4.1] (n = 40)
KL-6, U/ml	868.5 [547.3–1240.5] (n = 114)	503.0 [365.0–638.8] (n = 42)
SP-D ng/ml	203.0 [133–316] (n = 111)	186.0 [134.3–269.5] (n = 40)

BMI; body mass index, ESM_CSA_; cross-sectional area of elector spine muscles, ESM_MA_; muscle attenuation of elector spine muscles, FVC; forced vital capacity, FEV_1.0_; forced expiratory volume in 1.0 second, DLCO; diffuse capacity of the lung for carbon monoxide, KL-6; Krebs von den Lunge-6, SP-D; surfactant protein-D.

### Measurements of ESM_CSA_ and ESM_MA_

**Figure 1:**
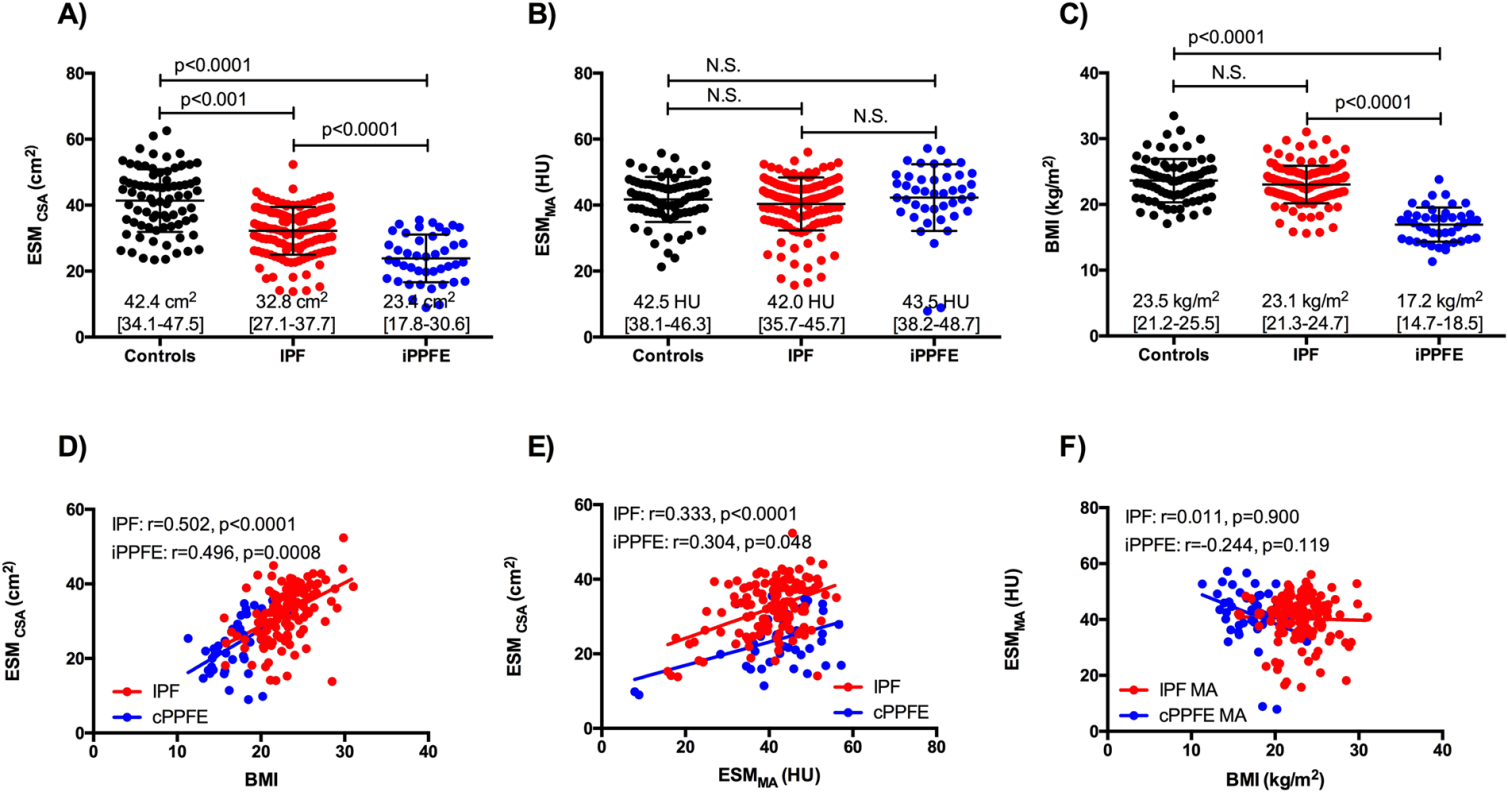
Prevalences of changes in body composition in patients with IPF and iPPFE. The distributions of ESM_CSA_ (**A**), ESM_MA_ (**B**), and BMI (**C**) in patients with IPF, iPPFE, and controls. Correlations between ESM_CSA_, ESM_MA_, and BMI in IPF, iPPFE. and controls (**D–F**).

The distributions of ESM_CSA_, ESM_MA_ and BMI are presented in Fig. 1A–C. The ESM_CSA_ of the patients with IPF and iPPFE were significantly smaller than that of control subjects (IPF; 32.8 [27.1–37.7] cm^2^, iPPFE; 23.4 [17.8–30.6] cm^2^, controls 42.4 [34.1–47.5] cm^2^; control vs IPF: p < 0.001, control vs iPPFE: p < 0.0001), and no difference was found in the ESM_MA_ between patients with ILDs and normal controls (IPF; 42.0 [35.7–45.7] HU, iPPFE; 43.5 [38.2–48.7] HU, controls; 42.5 [38.1–46.3] HU, respectively). BMI was significantly lower in the patients with iPPFE, but not in those with IPF compared to control subjects (IPF: 23.1 [21.3–24.7] kg/m^2^, iPPFE: 17.2 [14.7–18.5] kg/m^2^, controls: 23.5 [21.2–25.5] kg/m^2^; control vs IPF: p = 0.269, control vs iPPFE: p < 0.0001). Among the two ILDs, the ESM_CSA_ values and BMI were significantly lower in patients with iPPFE than in those with IPF (p < 0.0001, respectively), whilst ESM_MA_ did not differ significantly between the two groups.

Correlations between ESM_CSA_, ESM_MA_, BMI, and clinical parameters are shown in Fig. 1D–F, Tables 2 and 3. Significant correlations were found between ESM_CSA_ and BMI in patients with both IPF and iPPFE (r = 0.502, and r = 0.496); weaker correlations were found between ESM_CSA_ and ESM_MA_ (r = 0.333 and r = 0.304, respectively). ESM_MA_ did not correlate with BMI in patients with IPF or iPPFE. In the patients with both IPF and iPPFE, ESM_CSA_ correlated significantly with age and %FVC-predicted values; conversely, ESM_MA_ correlated with only age, but not pulmonary physiology data. ESM_CSA_ correlated weakly with smoking habit in patients with IPF, and with PaO_2_ in those with iPPFE.

**Table 2:** Correlations analyses of ESM_CSA_.

Variables	IPF (n = 131)		iPPFE (n = 43)	
	*r*	*P*-value	*r*	*P*-value
Age (yr)	−0.281	0.001	−0.291	0.058
BMI (kg/m^2^)	0.500	<0.0001	0.496	<0.0001
Smoking history (pack/year)	0.299	0.001	0.156	0.323
FVC (%)	0.389	<0.0001	0.438	0.007
FEV1/FVC (%)	−0.213	0.019	0.100	0.555
DLCO (%)	0.064	0.650	−0.025	0.907
PaO_2_ (Torr)	0.140	0.141	−0.381	0.018
PaCO_2_ (Torr)	−0.184	0.054	0.319	0.051
Alb (mg/dl)	0.119	0.187	0.283	0.077
KL-6 (U/ml)	0.064	0.501	0.190	0.227
SP-D (ng/ml)	−0.057	0.551	0.068	0.677

BMI; body mass index, FVC; forced vital capacity, FEV_1.0_; forced expiratory volume in 1.0 second, DLCO; diffuse capacity of the lung for carbon monoxide, KL-6; Krebs von den Lunge-6, SP-D; surfactant protein-D.

**Table 3:** Correlations analyses of ESM_MA_.

Variables	IPF (n = 131)		iPPFE (n = 43)	
	*r*	*P*-value	*r*	*P*-value
Age (yr)	−0.298	0.001	−0.312	0.042
BMI (kg/m^2^)	0.011	0.900	−0.244	0.119
Smoking history (pack/year)	0.008	0.929	0.017	0.913
FVC (%)	0.084	0.360	0.014	0.937
FEV1/FVC (%)	0.003	0.975	0.250	0.136
DLCO (%)	0.235	0.093	0.033	0.878
PaO_2_ (Torr)	0.142	0.138	−0.114	0.495
PaCO_2_ (Torr)	0.116	0.226	0.200	0.229
Alb (mg/dl)	0.023	0.800	0.174	0.283
KL-6 (U/ml)	−0.135	0.153	−0.038	0.810
SP-D (ng/ml)	−0.018	0.849	−0.168	0.301

BMI; body mass index, FVC; forced vital capacity, FEV_1.0_; forced expiratory volume in 1.0 second, DLCO; diffuse capacity of the lung for carbon monoxide, KL-6; Krebs von den Lunge-6, SP-D; surfactant protein-D.

### Prognostic value of ESM_CSA_ and ESM_MA_ in idiopathic pulmonary fibrosis and idiopathic pleuroparenchymal fibroelastosis

**Figure 2:**
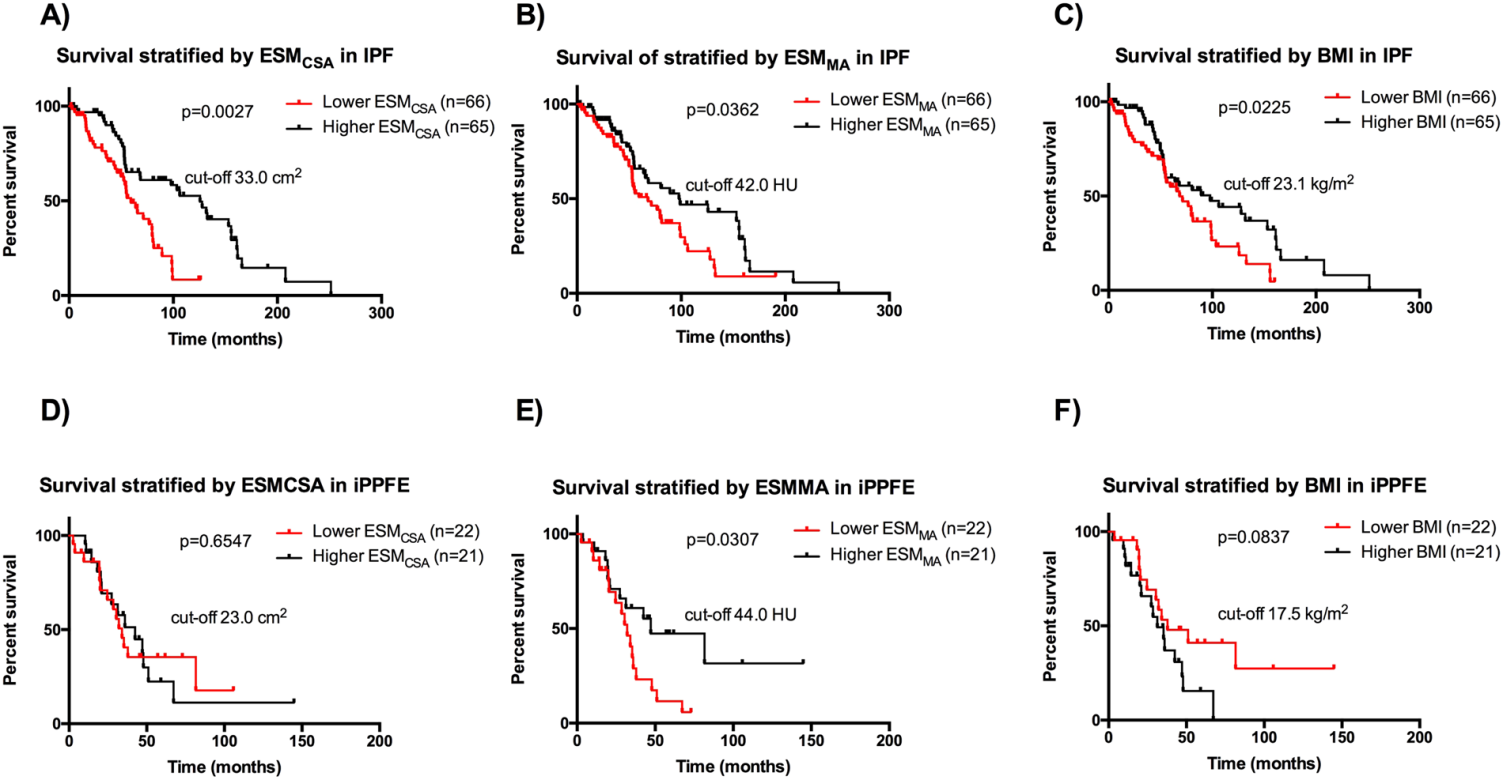
Prognostic impact of body composition changes in prognosis of IPF and iPPFE. Kaplan-Meier cureves of patienst with IPF and iPPFE according to ESM_CSA_ (**A**,**D**), ESM_MA_ (**B**,**E**), and BMI (**C**,**F**).

During follow-up (IPF: 53.3 [31.6–86.1] months, iPPFE: 31.3 [18.2–47.2] months, respectively), 76 patients with IPF and 27 with iPPFE died (causes of death in the patients with IPF included respiratory failure [n = 40], acute exacerbation [n = 21], cancer [n = 7], unknown cause [n = 6], sepsis [n = 1], and sudden death [n = 1]; causes of death in the patients with iPPFE included respiratory failure [n = 20], acute exacerbation [n = 5], hemoptysis [n = 1], and sudden death [n = 1]). We assessed the prognosis of IPF and iPPFE patients on either side of median ESM_CSA_, ESM_MA_ and BMI as a cut-off value using Kaplan-Meier method and log-rank test. In IPF, the patients with lower ESM_CSA_, ESM_MA_, and BMI values had significantly worse prognoses (p = 0.0027, p = 0.0362, p = 0.0225 respectively, Fig. 2A–C). In iPPFE, conversely, only ESM_MA_, but not ESM_CSA_ or BMI, effectively determined prognoses (Fig. 2D–F).

### Univariate and multivariate analyses of ESM_CSA_ and ESM_MA_ in idiopathic pulmonary fibrosis and idiopathic pleuroparenchymal fibroelastosis

To determine the impact of the measurements associated with body composition changes on prognosis, we preformed Cox proportion-hazard regression analyses. Univariate analyses in patients with IPF revealed that factors related with muscle wasting, such as ESM_CSA_, ESM_MA_, and BMI were significantly associated with mortality. Given that age and sex often deviate in accordance with body mass, we performed adjusted multivariate analyses with age and sex. We excluded DLCO, because it was examined in only 52 patients with IPF. In the multivariate analyses, both ESM_CSA_ and %FVC-predicted values were independently associated with mortality in patients with IPF (Table 4). In the iPPFE cohort, univariate analyses indicated only sex and ESM_MA_ as significant factors for mortality. Following adjustment for age and sex, lower ESM_MA_ values were associated with higher mortality rates in patients with iPPFE (Table 5).

**Table 4:** Prediction of Mortality in Patients with IPF by Univariate and Multivariate Cox-proportion Analyses.

Predictor	HR	95% CI	p-value	Predictor	HR	95% CI	p-value
**Univariate analysis**				**Multivariate analysis**			
Age, yr	1.039	1.012–1.068	0.005		1.033	0.996–1.070	0.089
Gender, female	1.622	0.796–3.306	0.183		0.757	0.332–1.725	0.508
BMI, kg/m^2^	0.880	0.804–0.964	0.006		1.009	0.892–1.141	0.890
ESM_CSA_, cm^2^	0.934	0.904–0.966	<0.001		0.951	0.906–0.998	0.042
ESM_MA_, (HU)	0.971	0.945–0.997	0.032		1.008	0.969–1.049	0.690
FVC, %-pred	0.956	0.938–0.974	<0.001		0.966	0.951–0.982	<0.001
FEV_1_/FVC, %	1.026	0.990–1.063	0.156				
DLCO, %	0.979	0.963–0.996	0.017				

BMI; body mass index, ESM_CSA_; cross-sectional area of elector spine muscles, ESM_MA_; muscle attenuation of elector spine muscles, FVC; forced vital capacity, FEV_1.0_; forced expiratory volume in 1.0 second, DLCO; diffuse capacity of the lung for carbon monoxide.

**Table 5:** Prediction of Mortality in Patients with iPPFE by Univariate and Multivariate Cox-proportion Analyses.

Predictor	HR	95% CI	p-value	Predictor	HR	95% CI	p-value
**Univariate analysis**				**Multivariate analysis**			
Age, yr	1.006	0.958–1.055	0.819		1.001	0.944–1.062	0.968
Gender, female	0.312	0.124–0.784	0.013		0.355	0.140–0.900	0.029
BMI, kg/m^2^	1.068	0.944–1.209	0.294				
ESM_/CSA_, cm^2^	0.972	0.918–1.029	0.323				
ESM_MA_, HU	0.953	0.920–0.987	0.008		0.959	0.926–0.994	0.023
FVC, %-pred	0.983	0.963–1.003	0.093				
FEV_1_/FVC, %	1.030	0.948–1.120	0.483				
DLCO, %	0.998	0.985–1.010	0.693				

BMI; body mass index, ESM_CSA_; cross-sectional area of elector spine muscles, ESM_MA_; muscle attenuation of elector spine muscles, FVC; forced vital capacity, FEV_1.0_; forced expiratory volume in 1.0 second, DLCO; diffuse capacity of the lung for carbon monoxide.

## Discussion

To our knowledge, this is the first study to evaluate body composition changes in patients with ILDs by means of quantitative measurement of ESM_CSA_, ESM_MA_, and BMI and to explore their clinical implications. First, we found lower ESM_CSA_, but not ESM_MA_, in patients with the ILDs versus controls. Among the ILDs, skeletal muscle loss in patients with IPF was not accompanied by weight loss, while patients with iPPFE showed more decline in skeletal muscle mass together with marked lean BMI values compared with those with IPF. Second, regarding the prognostic significance, a decline in ESM_CSA_ was associated with poor prognoses in patients with IPF, and this association was independent of established prognostic factors of IPF, such as age, sex, and pulmonary physiology. In contrast, lower ESM_MA_ values were independently related to poor survival in patients with iPPFE. These results suggest that distinct profiles of body composition changes occurred among IPF and iPPFE. More importantly, these changes were of prognostic significance, which highlights the importance of monitoring ESM_CSA_ and ESM_MA_ in the ILDs.

Loss of skeletal muscle, a hallmark of cachexia, is common in advanced lung disease. In addition, increased lipid deposition often occurs with declining muscle mass, and growing evidence supports an important role of fatty acid and intermediates in the regulation of muscle function^22^. Exposure to free fatty acids and excess dietary lipid intake are associated with pathogenesis of muscle wasting. Indeed saturated fatty acids convey detrimental effects upon muscle functions by causing insulin resistance, reactive oxygen species, inflammatory signals, and activate proteolysis, and also impairing protein synthesis and mitochondrial function^22^. Muscle mass loss can be assessed by measuring skeletal muscle cross-sectional area on CT, such as ESM_CSA_, whereas lipid deposition is evaluated by measures of skeletal muscle attenuation on CT, such as ESM_MA_^23,24^. When assessing cachexia, BMI does not accurately account for body composition changes and may underestimate the frequency of cachexia in obese patients; termed as sarcopenic obesity, or those who have gained weight due to edema^25,26^. Thus, quantitative analysis of body composition by ESM_CSA_ and ESM_MA_ is superior to BMI for assessing cachexia in COPD and cancer patients^12–14^. To date, however, no data are available on the assessment of body composition by ESM_CSA_ and ESM_MA_ in ILDs, such as IPF and iPPFE. Thus, in the present study, we attempted to investigate body composition changes determined by ESM_CSA_ and ESM_MA_ in patients with IPF and iPPFE. Compared with controls, ESM_CSA_ was significantly lower in patients with IPF and iPPFE, but ESM_MA_ was comparable. Among the measurements associated with body composition, ESM_CSA_ correlated fairly with BMI, and weakly with ESM_MA_. No significant correlation was found between ESM_MA_ and BMI. In addition, ESM_CSA_ correlated weakly with age and pulmonary physiology, whereas no correlation was observed between ESM_MA_ and pulmonary physiology. Collectively, these data suggest that each measurement may, in part, reflect different body composition changes and pathophysiology in ILDs. In cancer patients receiving chemotherapy, a significant decrease in both muscle CSA and attenuation on CT was reported, suggesting that these patients exhibited sarcopenic phenotype with decreased skeletal muscle mass and increased lipid deposition^27^. In COPD, however, although CSA of quadriceps was smaller in patients than in controls, muscle attenuation on CT did not differ significantly between them, which is similar to our observations in IPF and iPPFE^28^. These results suggest that patients with IPF and iPPFE may have decreased skeletal muscle mass without changes in lipid deposition.

Interestingly, a significant difference in body composition changes was found between IPF and iPPFE; lower ESM_CSA_ and smaller BMI values were noted in patients with iPPFE versus IPF, while ESM_MA_ values were similar in both groups. These data indicate that patients with iPPFE have greater loss of skeletal muscle mass, as well as a leaner body, compared to those with IPF, while patients with IPF show only moderate skeletal muscle loss without a change of BMI, suggesting different profiles of body composition changes between IPF and iPPFE. In our cohorts, patients with iPPFE were characterized by upper lobe predominant fibrosis together with more severe restrictive defects on spirometry than those with IPF. These differences might lead enhancements of impaired energy and protein balance, resulted in depletion of both fat and protein stores as reflected in weight loss and muscle wasting in iPPFE patients.

Most importantly, the present study indicates that the measurements associated with body composition were of prognostic significance, independent of age, sex, and pulmonary physiology. In IPF, patients with lower values of ESM_CSA_, ESM_MA_, and BMI had significantly worse survival than those with higher values. In iPPFE, patients with lower values of ESM_MA_ showed significantly poorer outcomes. Moreover, multivariate analyses with Cox proportional hazards regression model demonstrated that ESM_CSA_ and ESM_MA_ were independent prognostic factors for IPF and iPPFE, respectively. In patients with cancer, several studies reported that both skeletal muscle mass and its attenuation assessed by CT images were powerful prognostic factors, independent of BMI and clinical staging^12,29^. In COPD, ESM_CSA_ or CT-derived pectoralis muscle fat-free index correlated significantly with prognosis, independent of BMI and pulmonary physiology^13,14^. Thus, as observed in patients with cancer and COPD, the assessment of skeletal muscle by ESM_CSA_ and ESM_MA_ provides additional prognostic information in patients with IPF and iPPFE. Recently, several therapeutic molecules targeting for cachexia and muscle wasting, such as a ghrelin-receptor agonist (anamorelin) and a selective androgen receptor modulator (enobosarm), have been proven to be effective in patients with cancer^30,31^. Thus, it is possible that cachexia and muscle wasting may be a novel therapeutic target in ILDs.

Interestingly, as described above, we found a difference in prognostic factors associated with body composition changes between IPF and iPPFE. The reason for this difference is not clear. Generally, it is conceivable that ESM_CSA_ was a prognostic factor in IPF, because ESM_CSA_ has been reported to be of prognostic significance in other diseases, such as cancer and COPD, and patients with IPF had significantly lower ESM_CSA_ than controls. However, it is unclear why only ESM_MA_ had prognostic significance in iPPFE, despite no difference in ESM_MA_ between iPPFE and controls. In patients with advanced non-small cell lung cancer, Sjoblom and colleagues recently reported that lower skeletal muscle attenuation on CT was independently prognostic for poor survival, whereas cross-sectional muscle area was not^29^. This suggests that skeletal muscle adiposity may be more important than skeletal muscle loss in those patients for predicting prognosis. In addition, Maddocsks and colleagues showed that skeletal muscle attenuation in patients with COPD was not significantly different from that in controls, but the attenuation was associated with physical activity levels and exercise capacity in the patients^28^. In our patients with iPPFE, most of whom already had extremely low skeletal muscle mass, it is suggested that fat deposition assessed by ESM_MA_ may have been more closely associated with prognosis than skeletal muscle mass determined by ESM_CSA_. Future studies including larger patients with iPPFE will be required to confirm this.

The present study has several limitations. First, although a relatively large number of patients with IPF and iPPFE were enrolled, the data collection method was retrospective. Thus, the impact of longitudinal changes in weight loss, %FVC-predicted decline, ESM_CSA_, and ESM_MA_ were not evaluated. Additionally, we did not confirm our results among patients with pathologically diagnosed iPPFE. Although definite diagnosis of iPPFE requires pathological evaluation, surgical lung biopsies are not performed in a substantial number of cases in clinical practice due to the lack of curative treatment, limited ventilator reserve, risk of prolonged postoperative pneumothorax, and acute exacerbation^18^. Therefore, we have proposed clinical criteria for iPPFE, which enables us to recruit patients with characteristics similar to those of iPPFE^20^. Second, we measured ESM area at Th12, but did not evaluate the lumbar muscles or the pectoralis, which were explored in patients with COPD and cancer. Third, although D-XA is used to evaluate body composition changes, the associations between axial CT and D-XA measurements are unclear. Thus, future prospective studies are required to overcome these limitations.

In conclusion, the present study investigated body composition changes and their association with prognoses in patients with ILDs, including IPF and iPPFE. Compared with controls, distinct patterns of body composition changes occurred among patients with IPF and iPPFE, and the different measurements associated with body composition had prognostic significance for the two diseases. These results highlight the importance of assessing body composition changes in patients with ILDs. Additionally, management of muscle wasting may improve prognoses and provide a novel therapeutic target among these patients.

## Methods

### Subjects

This retrospective study was conducted on a cohort of 137 consecutive patients with IPF admitted to Hamamatsu University of School of Medicine between, and a cohort of 44 patients with clinically diagnosed PPFE admitted to the Hamamatsu University of School of Medicine and its nine associated hospitals between 2000 and 2015. Diagnosis of IPF was based on the ATS/ERS/Japanese Respiratory Society (JRS)/Latin American Thoracic Association (ALAT) criteria^17^. Clinical diagnosis of iPPFE was based on the following criteria^20^: (1) radiograph iPPFE pattern on chest CT (defined as bilateral subpleural dense consolidation with or without pleural thickening in the upper lobes, less marked according to Reddy’s radiological criteria^21^); (2) radiological confirmation of disease progression (defined as an increase in upper lobe consolidation with or without pleural thickening and/or a decrease in upper lobe volume on serial radiological assessment); (3) exclusion of other lung diseases with identifiable etiologies (such as connective tissue disease-related ILDs, chronic hypersensitivity pneumonitis, pulmonary sarcoidosis, pneumoconiosis, and active pulmonary infection). Chest CT at diagnosis was unavailable in 6 patients with IPF, and CT-imaging was insufficient for evaluation in one patient with iPPFE (due to spinal implants). Thus, this study enrolled 131 and 43 patients with IPF and iPPFE, respectively. As characteristics between IPF and iPPFE, such as gender, smoking habitant and body statures were different, this study also enrolled age matched 78 consecutive non-COPD subjects who visited our institute for medical check-up as controls; evaluations were performed using anthropometry, spirometry, and chest CT. None of the control subjects had ILDs, resected lung, active infections, malignancies, and neuromuscular diseases.

The study protocol was approved by the Ethical Committee of Hamamatsu University School of Medicine (17–196), and carried out in accordance with approved guideline. The need for patient approval and/or informed consent was waived due to the retrospective nature of the study.

### Computed-tomography image analysis

Electronically stored CT-images were used to assess muscle mass. All CT-images were obtained for diagnostic purposes during routine clinical practice. Chest CT was performed in the supine position at full inspiration breath-hold at 120 kVp and approximately 200 mA. Using a modified method, as described in a previous article^12,13,32^, chest CT images were reconstructed in a mediastinal setting (reconstruction kernel FC13). Single slice axial CT-images (contrast unenhanced condition; 5-mm thickness and 5-mm interval) taken at the lower margin of the 12th thoracic vertebra (Th12) were selected to measure ESM_CSA_. After imaging, ESMs were identified and manually shaded; ESM_CSA_ quantification was based on Hounsfield unit (HU) thresholds (−29 to + 150) and mean ESM_MA_ (HU) levels were assessed as previously described^12^. All CT analyses were independently performed by trained individuals (YS and KY) blinded to the patients’ survival statuses and then averaged. Images were analyzed using SYNAPSE VINCENT version 3 (FUJIFILM Medical Systems, Tokyo, Japan).

### Data collection

Clinical data were obtained from the patients’ medical records. Laboratory findings, pulmonary, and function test results, obtained at the time of diagnoses, were recorded.

### Statistical analysis

Discrete variables are expressed as totals (percentages), and continuous variables are expressed as median [interquartile range]. Mann-Whitney test was used to compare continuous variables, and the Kruskal-Wallis test and post hoc analyses were used for multiple comparisons. Fisher’s exact tests for independence were used to compare categorical variables. Correlations were analyzed using the Spearman’s rank correlation technique. Overall survival time was measured from date of IPF and iPPFE diagnosis. To determine the impact of body composition changes on prognoses, univariate and multivariate analyses were performed using the Cox proportional hazards regression model. Cumulative survival probabilities were estimated above and below the median ESM_CSA_, ESM_MA_, and BMI values using the Kaplan-Meier method and logrank test. Statistical analyses were performed using GraphPad Prism Version 6 (GraphPad Software, San Diego, CA, USA) and SPSS Statistics (Ver23, IBM Corporation, Armonk, NY, USA) software. All analyses were two-tailed and P values less than 0.05 were considered significant.

## Electronic supplementary material

Supplement Table1
