# Supplementary material for: Distinct profile and prognostic impact of body composition changes in idiopathic pulmonary fibrosis and idiopathic pleuroparenchymal fibroelastosis
Source: Sci Rep. 2018 Sep 19;8:14074. doi: 10.1038/s41598-018-32478-z (PMC6145941; doi:10.1038/s41598-018-32478-z)
Supplement: Supplementary file 1 — Supplement Table1 [file 41598_2018_32478_MOESM1_ESM.pdf]

Distinct profile and prognostic impact of body composition changes in idiopathic pulmonary fibrosis and idiopathic pleuroparenchymal fibroelastosis

Yuzo Suzuki, M.D., Ph.D.[1], Katsuhiro Yoshimura, M.D. [1], Yasunori Enomoto, M.D. [1], Hideki Yasui, M.D., Ph.D.[1], Hironao Hozumi, M.D., Ph.D.[1], Masato Karayama, M.D., Ph.D.[1], Kazuki Furuhashi, M.D., Ph.D.[1], Noriyuki Enomoto, M.D., Ph.D.[1], Tomoyuki Fujisawa, M.D., Ph.D.[1], Yutaro Nakamura, M.D., Ph.D.[1], Naoki Inui, M.D., Ph.D.[1], Takafumi Suda, M.D., Ph.D.[1]

[1]Second Division, Department of Internal Medicine, Hamamatsu University School of Medicine, Hamamatsu, Japan

Supplement Table 1. Clinical characteristics with 131 IPF patients, 43 iPPFE patients, and 78 controls.

	Controls (n=78)	IPF (n=131) UIP/IPF 50 (38.2%), cIPF 81(61.8%)	iPPFE (n=43)	*P*-value (IPF vs cPPFE)
Age, yr	70.0 [60.5-76.0]	69.0 [64.0-75.0]	69.0 [64.0-74.0]	p=0.863
Sex, male/female	62 (79.5%) / 16 (20.5%)	117 (89.3%) /14 (10.7%)	27 (62.8%) / 16 (37.2%)	p<0.001
Observation period, mo	–	53.3 [31.6-86.1]	31.3 [18.2-47.2]	p<0.001
Never or former/current smoker	1418 (18.0%), 64 (82.1%)	20 (15.3%), 111 (84.7%)	29 (67.4%), 14 (32.6%)	p<0.001
Smoking pack-year	29 [9.1-50.0]	35.0 [20.0-60.0]	0 [0-12.5]	p<0.001
acute exacerbation, yes	–	39 (29.8%)	8 (18.6%)	p=0.171
Height, cm	164.5 [159.1-169.2]	162.1 [157.0-166.0]	159.0 [152.0-165.0]	p=0.051
Weight, kg	63.9 [55.3-69.7]	60.0 [52.1-67.0]	42.3 [35.8-48.0]	p<0.001
BMI, kg/m^2	23.5 [21.2-25.5]	23.1 [21.3-24.7]	17.2 [14.7-18.5]	p<0.001
ESM_{CSA}, cm^2	42.4 [34.1-47.5]	32.8 [27.1-37.7]	23.4 [17.8-30.6]	p<0.001
ESM_{MA}, HU	42.5 [38.1-46.3]	42.0 [35.7-45.7]	43.5 [38.2-48.7]	p=0.090
**Pulmonary Function Test**				
FVC, %-pred	105.1 [92.0-113.1] (n=51)	80.5 [66.4-92.9] (n=120)	54.4 [45.8-67.5] (n=37)	p<0.001
FEV_1/FVC, %	77.3 [73.7-81.3] (n=51)	83.5 [79.4-88.0] (n=120)	96.3 [91.4-100] (n=37)	p<0.001
DLCO, %	–	68.6 [55.4-97.1] (n=52)	68.7 [47.9-91.9] (n=24)	p=0.700
**Laboratory**				
PaO_2, Torr	–	80.0 [73.0-89.1] (n=111)	79.0 [71.5-84.9] (n=38)	p=0.320
$PaCO_2$, Torr	–	41.9 [39.0-44.0] (n=111)	46.7 [41.3-49.0] (n=38)	p<0.001
Alb, g/dl	–	4.0 [3.8-4.3] (n=124)	4.0 [3.5-4.1] (n=40)	p=0.153

KL-6, U/ml	–	868.5 [547.3-1240.5] (n=114)	503.0 [365.0-638.8] (n=42)	p<0.001
SP-D ng/ml	–	203.0 [133-316] (n=111)	186.0 [134.3-269.5] (n=40)	p=0.565

BMI; body mass index, ESM_{CSA}; cross-sectional area of elector spine muscles, ESM_{MA}; muscle attenuation of elector spine muscles, FVC; forced vital capacity, $FEV_{1.0}$; forced expiratory volume in 1.0 second, DLCO; diffuse capacity of the lung for carbon monoxide, KL-6; Krebs von den Lunge-6, SP-D; surfactant protein-D
